# Association of Polo-Like Kinase 3 and PhosphoT273 Caspase 8 Levels With Disease-Related Outcomes Among Cervical Squamous Cell Carcinoma Patients Treated With Chemoradiation and Brachytherapy

**DOI:** 10.3389/fonc.2019.00742

**Published:** 2019-08-14

**Authors:** Max Fleischmann, Daniel Martin, Samuel Peña-Llopis, Julius Oppermann, Jens von der Grün, Markus Diefenhardt, Georgios Chatzikonstantinou, Emmanouil Fokas, Claus Rödel, Klaus Strebhardt, Sven Becker, Franz Rödel, Nikolaos Tselis

**Affiliations:** ^1^Department of Radiotherapy and Oncology, Goethe-University, Frankfurt, Germany; ^2^Division of Solid Tumor Translational Oncology, West German Cancer Center, Essen University Hospital, Essen, Germany; ^3^German Cancer Research Center (DKFZ), Heidelberg, Germany; ^4^German Cancer Consortium (DKTK) Partner Site, Essen/Düsseldorf, Germany; ^5^Frankfurt Cancer Institute (FCI), University of Frankfurt, Frankfurt, Germany; ^6^German Cancer Consortium (DKTK) Partner Site, Frankfurt/Mainz, Germany; ^7^Department of Gynecology, Goethe-University, Frankfurt, Germany

**Keywords:** cervical cancer, polo-like kinase 3, caspase 8, chemoradiotherapy, local control, cancer-specific survival, overall survival

## Abstract

**Introduction:** Definitive chemoradiation (CRT) followed by high-dose-rate (HDR) brachytherapy (BT) represents state-of-the-art treatment for locally-advanced cervical cancer. Despite use of this treatment paradigm, disease-related outcomes have stagnated in recent years, indicating the need for biomarker development and improved patient stratification. Here, we report the association of Polo-like kinase (PLK) 3 expression and Caspase 8 T273 phosphorylation levels with survival among patients with cervical squamous cell carcinoma (CSCC) treated with CRT plus BT.

**Methods:** We identified 74 patients with FIGO Stage Ib to IVb cervix squamous cell carcinoma. Baseline immunohistochemical scoring of PLK3 and pT273 Caspase 8 levels was performed on pre-treatment samples. Correlation was then assessed between marker expression and clinical endpoints, including cumulative incidences of local and distant failure, cancer-specific survival (CSS) and overall survival (OS). Data were then validated using The Cancer Genome Atlas (TCGA) dataset.

**Results:** PLK3 expression levels were associated with pT273 Caspase 8 levels (*p* = 0.009), as well as N stage (*p* = 0.046), M stage (*p* = 0.026), and FIGO stage (*p* = 0.001). By the same token, pT273 Caspase 8 levels were associated with T stage (*p* = 0.031). Increased PLK3 levels corresponded to a lower risk of distant relapse (*p* = 0.009), improved CSS (*p* = 0.001), and OS (*p* = 0.003). Phospho T273 Caspase 8 similarly corresponded to decreased risk of distant failure (*p* = 0.021), and increased CSS (*p* < 0.001) and OS (*p* < 0.001) and remained a significant predictor for OS on multivariate analysis. TCGA data confirmed the association of low PLK3 expression with resistance to radiotherapy and BT (*p* < 0.05), as well as increased propensity for metastasis (*p* = 0.019). Finally, a combined PLK3 and pT273 Caspase 8 score predicted for decreased distant relapse (*p* = 0.005), and both improved CSS (*p* < 0.001) and OS (*p* < 0.001); this combined score independently predicted distant failure (*p* = 0.041) and CSS (*p* = 0.003) on multivariate analyses.

**Conclusion:** Increased pre-treatment tumor levels of PLK3 and pT273 Caspase 8 correspond to improved disease-related outcomes among cervical cancer patients treated with CRT plus BT, representing a potential biomarker in this context.

## Introduction

As a result of screening regimens in the developed world, the incidence of uterine cervical cancer has been relatively constant in recent years, with an estimated 4,540 cases and 1,506 deaths from cervical cancer in Germany in 2014 (1). However, the entity still comprises the fourth most frequently occurring malignancy in women worldwide (2, 3). Definitive CRT followed by HDR-BT is currently the standard-of-care therapy for patients with locally advanced disease, generally including those patients with at least International Federation of Gynecology and Obstetrics (FIGO) stage IIb lesions. In the modern era, local control for such patients with locally-advanced disease ranges between 74 and 85% (4). Moreover, for all stages, the 3-year survival rate in developed countries is 68% (4) and <50 % in non-developed countries (5). Nevertheless, survival rates were stagnating in recent years and effective treatment strategies for locally recurrent or metastatic disease still remain elusive indicating the need for biomarker development and translational research (6).

Polo like kinase 3 (PLK3), identified and cloned by our group (7) is a member of PLK family that encompasses five mammalian enzymes (PLK1, PLK2/Snk, PLK3/Fnk/Prk, PLK4/Sak, and PLK5) which regulate multiple components of cell cycle progression, entry into mitosis, and replication of DNA (8–10). A common feature of PLK1-PLK4 is a N-terminal serine/threonine kinase domain and a regulatory Polo-Box-Domain (PBD) in the C-terminus which is involved in subcellular localization, enzymatic activity, and substrate interaction (11). In malignant cells, PLK activity is dysregulated, resulting in increased proliferation, as well as invasion and resistance to apoptosis (12–14). Accordingly, a molecular targeting of members of the PLK family may represent an efficacious oncologic approach. This is supported by data demonstrating improved survival among acute myeloid leukemia patients treated with a PLK1 small molecular inhibitor BI 6727 (Volasertib) (15).

While the prognostic impact of PLK1 has been demonstrated across several disease sites [reviewed in (9, 12)], there is controversy regarding the prognostic value of PLK3 expression. Despite the fact that PLK3 overexpression is correlated with unfavorable outcomes in ovarian, breast, and prostate carcinomas (16–18), increased expression of PLK3 is associated with improved outcomes in hepatocellular cancer, lung cancer and human papilloma virus (HPV) associated lesions including head-and-neck as well as anal carcinoma (19–22).

Upon oxidative stress or DNA damage PLK3 kinase activity is increased in dependence of the Ataxia telangiectasia mutated (ATM) kinase and results in a phosphorylation of TP53 and checkpoint kinase 2 (Chk2) linking DNA damage to cell cycle arrest and apoptotic cell death (10, 23). Further, PLK3 directly impacts on DNA double-strand break repair by phosphorylation of C-terminal binding protein (CtbP) interaction protein (CtIP) initiating microhomology-mediated end joining (MMEJ) (24, 25). We have recently reported PLK3 phosphorylation of pro-Caspase 8 at residue T273 to promote apoptosis (26). Caspase 8 is a central component of the extrinsic apoptotic pathway (27) and thus, T273 phosphorylation by PLK3 serves to promote cell death (26). Clinically, we recently further reported that low levels of pT273 Caspase 8 and PLK3 in anal tumors are associated with inferior disease control (both local and distant), as well as poorer survival, following definitive CRT (22). Notably, in this tumor entity for both PLK3 and pT273 caspase-8 signals a significant positive correlation to the extent of HPV infection was evident (22) with quantitative HPV viral load and p16^INK4a^ expression to further correlate to local control as well as patients overall survival (28).

Here, we describe the association between disease-related outcomes and pre-treatment tumor PLK3 and pT273 Caspase 8 detection among locally-advanced cervical cancer patients.

## Materials and Methods

### Patient Characteristics

We identified, through retrospective review, 74 patients with uterine cervix squamous cell carcinoma treated with definitive CRT between 1999 and 2017 at our institution. Eligibility criteria included histological proof of cervix carcinoma FIGO stages Ib to IVb (29) and curative intent of CRT/BT. Patients were routinely subjected to standard pre-treatment staging including computer tomography or magnetic resonance tomography of the pelvis and abdomen, chest radiography, and baseline laboratory studies. Institutional review board approval was obtained in accordance with the Helsinki Declaration of 1975.

### Treatment and Follow-Up Assessment

Treatment covered concomitant radiotherapy by photon beam linear accelerators (Elekta, Crowley, UK) followed by intracavitary +/– interstitial HDR-BT. Pelvic external beam radiotherapy (EBRT) was administered using a conventional four-field technique (*n* = 22), intensity-modulated RT (IMRT) or 3-D conformal RT using high-energy photons (*n* = 52). Median EBRT dose was 50.4 Gy (range, 45.0–66.6 Gy) delivered in daily 1.8 Gy fractions (five fractions per week). Median physical BT dose was 40 Gy (range, 4.0–48.0 Gy). The median overall EQD2 generated from EBRT + BT for all patients amounted to 106.2 Gy (range, 54.2–121.6 Gy). A minimum EQD2 of 78.1 Gy was applied to every patient, who completed therapy. Cisplatin-based chemotherapy was administered weekly (40 mg/m^2^) or in the first and last week of treatment concurrent to EBRT (20 mg/m^2^/day). Twelve patients additionally received two cycles of 5-Fluorouracil at 600 mg/m^2^, two patients Mitomycin-C at 7 mg/m^2^ or Paclitaxel at 25 mg/m^2^. One patient received Cisplatin in combination with Gemcitabine (750 mg/m^2^). Follow-up examinations occurred every 3 months during the first 24 months after CRT, and every 6 months thereafter. Monitoring included gynecological examination and imaging with pelvic MRI and/or abdominal CT.

### Immunohistochemical Staining and Scoring

Pre-treatment formalin fixed paraffin embedded (FFPE) biopsy tissues were stained manually by an experienced technician using DAKO EnVisionTM FLEX Peroxidase Blocking reagent (K8000, DAKO, Hamburg, Germany). Primary anti-PLK3 (1:50 dilution, ab33119, Abcam, Cambridge, UK), anti-pT273 caspase 8 antibodies established by our group (26) at a 1:200 dilution and anti p16^INK4a^ (CINtec Kit, Roche, Basel, Switzerland) were applied for 120 min at room temperature. Next, samples were incubated for 60 min with anti mouse and rabbit secondary antibodies (SM802) derived from a DAKO EnVision™ FLEX kit (K8000, DAKO) and epitope-antibody products were visualized using dextran polymer conjugated horseradish peroxidase as well as 3,3′-diaminobenzidine (DAB) chromogen. Counterstaining was performed using haematoxylin (Gill 3, Sigma Aldrich, Munich, Germany). Appropriate negative controls were utilized, and stained in the absence of the corresponding primary antibody. PLK3 expression was dichotomized as being “high” (weighted score [WS] > 6) or “low” (≤6) based on a combination of considering the fraction of PLK3-positive tumor cells [1: (0–25%), 2: (26–50%), 3: (51–75%), and 4: (>75%)] as well as the intensity of immunohistochemical PLK3 staining (1: weak, 2: moderate, and 3: intense) as previously reported (22).

T273 Caspase 8 phosphorylation was scored as a percentage of the pT273 Caspase 8 positive tumor cells as noted via immunohistochemistry. Quantification of histochemical p16^INK4a^ detection with WS defined as for PLK3 has been previously reported in detail (28). Immunohistochemical samples were independently evaluated by two investigators (MF, FR) who were blinded to patient-specific clinical information. Image acquisition was via AxioImager Z1 microscope, with an Axiocam camera and associated Axiovision 4.6 software (Zeiss, Göttingen, Germany).

### Cervical Cancer TCGA Datasets

RNA-Sequencing (RNA-Seq) and associated clinical data for CSSC and endocervical adenocarcinoma (CESC) patients were downloaded from The Cancer Genome Atlas (TCGA) (https://portal.gdc.cancer.gov/). Genomic clinical associations were performed as previously reported (30). Briefly, gene expression normalization was performed using the RNA-Seq Expectation-Maximization (RSEM) method. Gene expression was then stratified by quartiles, where the 1st quartile represented the lowest-expression, the 2nd and 3rd quartiles an intermediate expression and the 4th quartile the highest expression. To compute overall survival, we considered the patient date of death or the last date the patient was known to be alive (last follow-up). Resistance to EBRT, BT, and chemotherapy was defined as progressive disease or partial response compared to patients that displayed complete responses.

### Statistical Analysis

Correlation of pathologic factors, including immunohistochemical scoring, was performed with Spearman's correlation coefficient. Local control was defined from the time of CRT initiation, with first local tumor detection or progression after response following CRT scored as a local failure event. Cancer-specific survival (CSS) and overall survival (OS) were defined from CRT initiation; cancer-related death represented a CSS event. Survival curves for all disease-related outcomes were calculated per the Kaplan-Meier method; log-rank testing and Cox proportional hazard modeling were utilized for univariate and multivariate survival analyses, respectively. Statistical tests were performed with *a priori* α = 0.05 for significance; all tests were performed using IBM SPSS Version 25 Software (IBM, Ehningen, Germany).

## Results

### Tumor and Patient Histopathologic Characteristics

**Figure 1:**
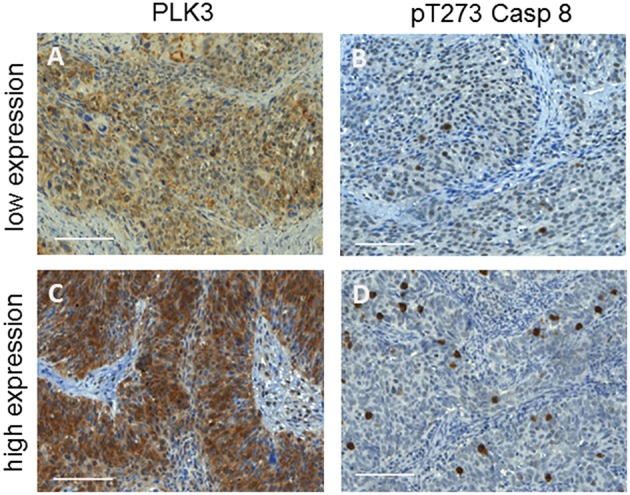
Examples of cervix cancer biopsies with a low **(A,B)** or high **(C,D)** immuno-histochemical detection of PLK3 and pT273 caspase 8. Original magnification × 40, scale bars: 100 μm.

Assessing pre-treatment tumor samples, 54 patients (73%) presented with increased PLK3 expression, 33 (44.6%) with increased pT273 Caspase 8 levels, and 47 (63.5%) with high p16^INK4a^ detection (Table 1). Figure 1 highlights examples of dichotomized staining for PLK3 and pT273 Caspase 8 immunohistochemistry.

**Table 1:** Results of PLK3, pT273 caspase 8, and p16^INK4a^ immunohistochemistry.

**Marker**	**PLK3 *n* (%)**	**pCasp 8 *n* (%)**	**p16^**INK4a**^*n* (%)**
Dichotomized score	≤6 WS >6	≤ median >	≤6 WS >6
Low score	20 (27.0)	41 (55.4)	27 (36.5)
High score	54 (73.0)	33 (44.6)	47 (63.5)

*Dichotomized score low vs. high based on the median weighted score (WS: intensity x % pos. tumor cells), median pT273 Casp8 positive cells (%)*.

CSCC is considered to be associated with a high prevalence of HPV DNA detection and surrogate marker p16^INK4a^ expression (31, 32). We have recently shown that quantitative HPV viral load and p16^INK4a^ expression significantly correlated with local control as well as overall survival among anal cancer patients (28). Thus, we wanted to assess the predictive value of quantitative p16^INK4a^ scoring on clinical endpoints in our patient cohort. We observed a significant impact of quantitative p16^INK4a^ detection on local failure (*p* = 0.014) and CSS (*p* = 0.013), and a trend for OS (*p* = 0.061), while distant failure was not correlated to p16^INK4a^ expression (*p* = 0.346, Supplemental Figure 1).

Elevated PLK3 expression was correlated with elevated pT273 Caspase 8 levels (*p* = 0.009). By contrast, we did not detect a significant relationship to p16^INK4a^ expression for both, PLK3 and pT273 caspase 8 expression (Table 2). Concerning patient- and tumor-related characteristics, elevated levels of PLK3 detection were more prevalent in patients with lower N-stage (*p* = 0.046), M-stage (*p* = 0.026), and FIGO category (*p* = 0.001), while no significant differences for age, T category and tumor grading were observed (Table 2). Phospho T273 caspase 8 detection was significantly (*p* = 0.031) associated with T stage, but not with age, N and M stage, FIGO category, and tumor grading (Table 2).

**Table 2:** Clinicopathological findings according to PLK3 expression and T273 Caspase 8 phosphorylation.

	**No. of patients**	**PLK3 WS ≤ 6 *n* (%)**	**PLK3 WS > 6 *n* (%)**	***p*-value**	**pCasp 8 ≤ Med *n* (%)**	**p Casp 8 > Med *n* (%)**	***p*-value**
**AGE**							
≤59 years	38	12 (16.2)	26 (35.1)		17 (23.0)	21 (28.4)	
>59 years	36	8 (10.8)	28 (37.8)	0.372	24 (32.4)	12 (16.2)	0.059
**T-STAGE**							
T1/2	39	7 (9.5)	32 (43.2)		17 (23.0)	22 (29.7)	
T3/4	35	13 (17.6)	22 (29.7)	0.065	24 (32.4)	11 (14.9)	**0.031**
**N-STAGE**							
N0	39	7 (9.5)	32 (43.2)		22 (29.7)	17 (23.0)	
N1	34	12 (16.2)	22 (39.7)		19 (25.7)	15 (20.3)	
Nx	1	1 (1.4)	0	**0.046**	0	1 (1.4)	0.766
**M-STATUS**							
M0	63	14 (18.9)	49 (66.2)		33 (44.6)	30 (40.5)	
M1	11	6 (8.1)	5 (6.8)	**0.026**	8 (10.8)	3 (4.1)	0.216
**FIGO**							
Low (Ia-IIb)	29	2 (2.7)	27 (36.5)		13 (17.6)	16 (21.6)	
High (IIIa-IVb)	45	18 (24.3)	27 (36.5)	**0.001**	28 (37.8)	17 (23.0)	0.146
**GRADING**							
G1/2	38	7 (9.5)	31 (41.9)		18 (24.2)	20 (27.0)	
G3	34	13 (17.6)	21 (28.4)	0.135	23 (31.1)	11 (14.9)	
Gx	2		2 (2.7)			2 (2.7)	0.267
**P16**							
Low (WS ≤ 6)	27	9 (12.2)	18 (24.3)		19 (25.7)	8 (10.8)	
High (WS > 6)	47	11 (14.9)	36 (48.6)	0.361	22 (29.7)	25 (33.8)	0.051
**PLK3**							
Low (WS ≤ 6)	20				16 (21.8)	4 (5.4)	
High (WS > 6)	54				23 (33.8)	29 (39.2)	**0.009**
**pCasp 8**							
Low (≤ Median)	41	16 (21.8)	25 (33.8)				
High (> Median)	33	4 (5.4)	29 (39.2)	**0.009**			

*Significant values are given in bold*.

### Disease-Related Outcomes

**Figure 2:**
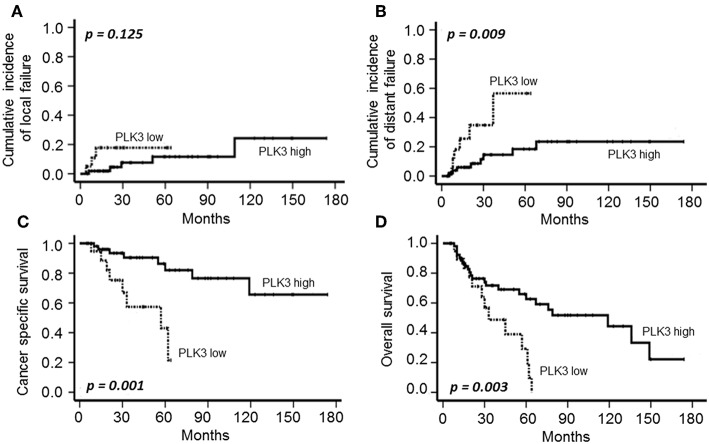
Cumulative incidence of local **(A)** and distant failure **(B)**, CSS **(C)** and OS **(D)** according to PLK3 expression (low: individual WS ≤ 6; high: individual WS > 6) in patients with CSCC treated with definitive CRT and BT.

**Figure 3:**
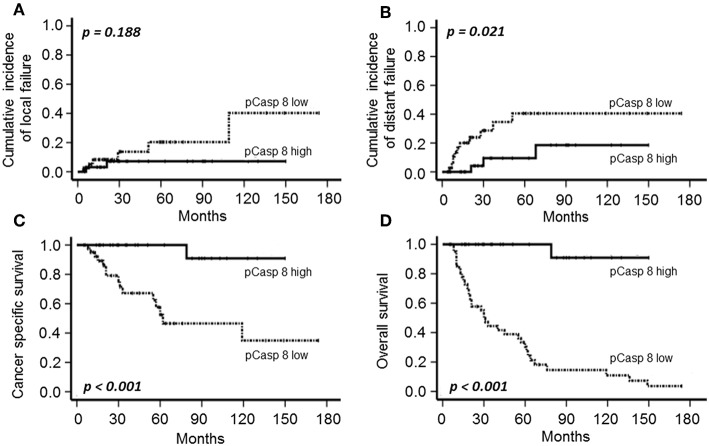
Cumulative incidence of local **(A)** and distant failure **(B)**, CSS **(C)** and OS **(D)** according to T273 Caspase 8 phosphorylation (low: individual WS≤median; high: individual WS>median) in patients with CSCC treated with definitive CRT and BT.

Following definitive CRT plus BT, 19 patients (25.6 %) experienced disease relapse, including local recurrence (*n* = 8) and/or distant metastasis (*n* = 15). Local failure was associated with both T-stage (*p* = 0.011) and FIGO category (*p* = 0.006) in univariate analysis, while PLK3 and pT273 caspase 8 did not significantly impact on local control (Figures 2A, 3A).

The cumulative 5- and 10-year distant metastasis incidence in the cohort were estimated at 22.3 and 29.8%, respectively. T-stage (*p* = 0.011), FIGO category (*p* = 0.008), low PLK3 (Figure 2B, *p* = 0.009), and low pT273 caspase 8 levels (Figure 3B, *p* = 0.021) were associated with distant metastasis risk; only FIGO stage continued to predict for distant metastasis risk on multivariate analysis (*p* = 0.041) (Table 3).

**Table 3:** Univariate and multivariate analyses of prognostic factors in patients with CSCC.

			**Multivariate analyses**		
			**95 % confidence interval (CI)**		
	**Univariate *p*-value**	**Hazard ratio (HR)**	**Lower**	**Upper**	***p*-value**
**CUMULATIVE INCIDENCE OF LOCAL FAILURE**					
T-stage (T1-2/T3-4)	**0.011**	1.51	0.29	7.90	0.619
FIGO (Ia-IIb/IIIa-IVb)	**0.006**	5.07	0.62	41.23	0.129
**CUMULATIVE INCIDENCE OF DISTANT FAILURE**					
T-stage (T1-2/T3-4)	**0.011**	1.16	0.24	5.66	0.850
FIGO (Ia-IIb/IIIa-IVb)	**0.008**	8.40	1.19	64.75	**0.041**
PLK3 (WS ≤ 6/> 6)	**0.009**	1.87	0.57	6.12	0.299
pCasp 8 (≤/> median)	**0.021**	3.18	0.88	11.52	0.077
**CANCER-SPECIFIC SURVIVAL**					
T-stage (T1-2/T3-4)	**0.006**	2.15	0.056	8.24	0.263
FIGO (Ia-IIB/IIIa-IVb)	**0.017**	1.55	0.12	19.05	0.732
p16 (WS ≤ 6/>6)	**0.013**	2.43	0.86	6.70	0.094
PLK3 (WS ≤ 6/>6)	**0.001**	2.89	0.94	8.83	0.062
pCasp 8 (≤/> median)	** <0.001**	9.12	1.10	75.14	**0.040**
**OVERALL SURVIVAL**					
PLK3 (WS ≤ 6/>6)	**0.003**	1.39	0.66	2.90	0.377
pCasp 8 (≤/> median)	** <0.001**	35.94	4.90	263.47	** <0.001**

*Significant values are given in bold*.

With a median of 32 months follow-up (range: 5–174 months), OS and CSS were 51.4 and 78.4%, respectively. A total of 36 patients died during follow-up; 16 due to cervical cancer, 16 of intercurrent diseases and three because of treatment-related events. Clinical factors with a significant impact on the oncologic outcome were T- and FIGO-stage on CSS (*p* = 0.006 and *p* = 0.017). As shown in Figures 2C, 3C, both PLK3 expression (*p* = 0.001) and pT273 caspase 8 levels (*p* < 0.001) were associated with CSS on univariate analysis; this was true for both markers with regard to OS as well (PLK3, *p* = 0.003, Figure 2D; pT273 Caspase 8, *p* < 0.001, Figure 3D). On multivariate analyses, pT273 caspase 8 phosphorylation remained a significant independent predictor for both CSS (*p* = 0.04) and OS (*p* < 0.001) (Table 3).

**Figure 4:**
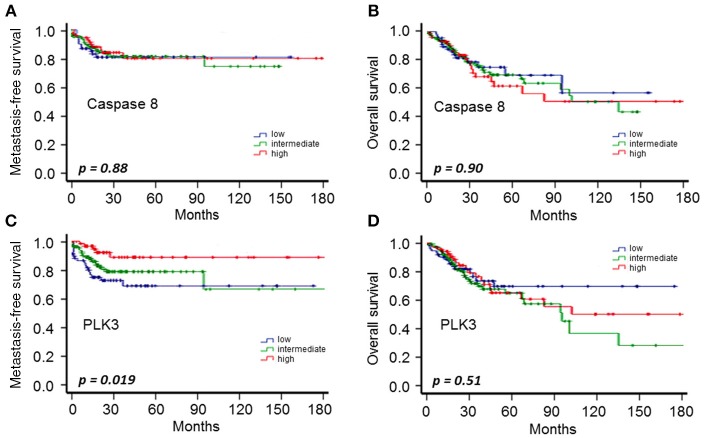
CESC-TCGA data on metastasis-free survival **(A,C)** and OS **(B,D)** according to PLK3 and Caspase 8 gene expression (low: 1st quartile; intermediate: 2nd and 3rd quartiles; high: 4th quartile) in CESC patients from TCGA.

To validate these findings regarding the prognostic role of PLK3, we analyzed TCGA data from cervical cancer (CESC) patients and while there were no differences in metastasis and overall survival for Caspase 8 gene expression and OS for PLK3 gene expression, we found a significant association between low PLK3 gene expression and poor metastasis-free survival (*p* = 0.019) (Figure 4). Notably, patients with low expression levels of PLK3 indicated a significant (*p* < 0.05) association with resistance to EBRT and BT, but not to chemotherapy (Table 4). Caspase 8 mRNA expression had no impact on these properties.

**Table 4:** Development of resistance according to PLK3 and Caspase 8 gene expression.

**Patient resistance**		***n***	***CASP 8***	***PLK3***
Chemoresistant	No	53	618 ± 32	475 ± 68
	Yes	24	597 ± 63	472 ± 53
Radioresistant	No	115	593 ± 23	515 ± 42
	Yes	31	606 ± 54	390 ± 36^*^
Brachyresistant	No	65	576 ± 30	488 ± 46
	Yes	19	646 ± 69	359 ± 43^*^

Average ± SEM;

* *P < 0.05*.

*Data represent RNA-Seq Expectation-Maximization (RSEM) normalized expression values*.

**Figure 5:**
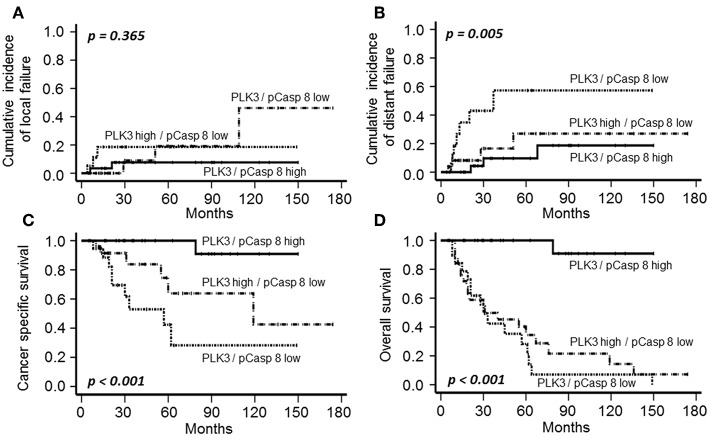
Cumulative incidence of local **(A)** and distant failure **(B)**, CSS **(C)**, and OS **(D)** according to PLK3 and T273 Caspase 8 phosphorylation (high PLK3 and high pCasp 8 vs. high PLK3 and low pCasp 8 vs. low PLK3 and low pCasp 8) in patients with CSCC treated with definitive CRT and BT.

Finally, given the direct correlation between PLK3 and pT273 Caspase 8 levels as shown above (*p* = 0.009), we combined these molecular features into a single variable. A combined PLK3 (>WS6) and pT273 caspase 8 (>Median) variable was significantly associated with disease-related outcome, including distant failure (*p* = 0.005), CSS (*p* < 0.001), and OS (*p* < 0.001) (Figure 5). Multivariate analysis confirmed the independent predictive capacity of this combined variable for distant failure (*p* = 0.041) and CSS (*p* = 0.003) (Supplemental Table 1).

## Discussion

At present, only a few valid markers to predict outcome and survival of patients with uterine cervical cancer patients are available. In particular, FIGO stage, primary tumor size, parametrial, and lymph node invasion, as well as the quality and availability of BT are considered essential and critical parameters in predicting treatment success (33). Moreover, overexpression of a variety of molecular (bio)markers including among others epidermal growth factor receptor has been shown to be associated with poor response to CRT, poor disease-free and OS [reviewed in (34)]. The prognostic impact of PLK3 and its substrate pT273 Caspase 8, however, has not been evaluated in cervical cancer thus far to the best of our knowledge.

Notably, there are controversial reports on the prognostic impact of PLK3 in different tumor entities. Whereas, overexpression of PLK3 has been correlated with a non-favorable outcome in ovarian, breast, and prostate cancer (16–18), contradicting evidence exists in hepatocellular carcinoma, in lung cancer, in head and neck squamous cell and in anal squamous cell carcinoma, where PLK3 is considered to be a tumor suppressor and is correlated with an improved tumor control and survival (19–22). In line with these latter findings, we indicate a high expression of PLK3 to significantly correlate with improved distant tumor control and long-term survival (Figure 2) in a CSCC cohort treated with definitive CRT plus BT, supporting its proposed tumor suppressor activity.

The underlying basis for increased PLK3 expression being associated with improved patient survival remains elusive. It is reasonable to speculate that this relationship may be a function of DNA damage response and apoptosis induction (10, 23, 35). Concerning DNA damage response, PLK3 mediates a response pathway involving ATM as well as TP53 (35, 36). PLK3 further interacts with Chk2 (37) and mediates Cdc25 phosphatase function, linking oxidative stress and DNA damage to G1-S transition and cell cycle arrest (10). Furthermore, PLK3 directly impacts on DNA double-strand break repair by phosphorylation of CtIP in G1, which promotes the resection of DNA double-strand breaks to initiate alternative repair pathways like MMEJ (24, 25). Additionally, PLK3 induction by transcription factor nuclear factor kappa B activity (37) indicates a pro-apoptotic activity of the kinase. Our group has previously demonstrated that T273 phosphorylation of Caspase 8 by PLK3 enhances its pro-apoptotic function, likely through the extrinsic apoptotic pathway (26).

Caspase 8 is involved in death induction signaling as well as necroptosis, extracellular matrix adhesion, and cell migration (38–42). With this in mind, Caspase 8 may also affect cancer cell metastatic potential and behavior. This is supported by our data demonstrating an inverse relationship between pT273 Caspase 8 levels (as well as PLK3 levels) with distant metastasis incidence, where lower pT273 Caspase 8 levels, for instance, predict increased risk of distant relapse.

CSCC is a prime example of a malignancy associated with a high prevalence of HPV DNA and surrogate marker p16^INK4a^ detection which are associated with an improved tumor remission and prognosis following RT or CRT (31, 32, 43). By applying a quantitative assessment in our patient cohort, we observed a significant impact of a high p16^INK4a^ expression on an improved local failure, CSS and a trend for OS, while distant failure was not correlated to p16^INK4a^ (Supplemental Figure 1) confirming the marker to display a positive prognosticator for increased local and tumor response. However, in contrast to a recent investigation in anal carcinoma treated with definitive RCT (22), we did neither recognize a significant correlation of p16^INK4a^ with PLK3 and a borderline significance with pT273 Caspase 8 expression (Table 2), nor did we observe a significant impact of PLK3 and pT273 Caspase 8 detection on the incidence of local failure (Figures 2, 3). There are different aspects to explain this discrepancy. First, in contrast to anal cancer local failure is a prescriptive/narrow and deliberately confined event in recurrent cervical cancer. Local recurrence is defined and restricted as a central or pelvic side wall recurrence, while relapsing at para-aortic or inguinal lymph nodes already is defined as metastatic disease. Second, as compared to anal carcinoma, treatment regimens in cervical cancer differ by the addition of BT as an integral component to maximize the probability of achieving local control. Quality and availability of BT, however, are critical parameters in predicting treatment success (44).

Limitations of our study include caveats conventionally associated with a retrospective series involving a relatively modest pool of patients and few events. The retrospective design of our analysis is more susceptible to election, misclassification, and therefore calculation bias. While confirmation of our findings in a larger cohort of patients is indicated, we endeavored to address this limitation in some respect through analysis of TCGA data as an independent validation cohort. CASP8 gene expression showed no clinical significance for OS or incidence of metastasis, not discarding that posttranscriptional modification of Caspase 8 might be more relevant clinically. PLK3 gene expression instead showed that, although not associated with OS, patients with low PLK3 expression levels presented higher incidence of metastasis. In addition, low PLK3 expression was correlated with resistance to EBRT and BT, but not chemotherapy, in cervical cancer patients, again confirming the association of PLK3 levels with prognosis.

In summary, our data support the notion that increased pre-treatment tumor levels of PLK3 and pT273 caspase 8 predict for superior clinical response among cervical cancer patients treated with definitive CRT plus BT. Beyond the association of these markers with clinical outcomes, these results may impact on therapeutic decisions. Existing PLK1 small molecular inhibitors may exhibit PLK3 cross-reactivity and therefore may also contribute to PLK3 inhibition (12). In light of the hypothesized tumor suppressive role of PLK3 in cervical cancer, we advise caution in future clinical investigations regarding the utility of PLK1 inhibitors in this setting.

## Data Availability

All datasets generated for this study are included in the manuscript and/or the Supplementary Files.

## Author Contributions

MF, DM, SP-L, CR, KS, JvdG, MD, GC, and SB provided patient data and material. JO contributed to the sample-preparation and staining. MF, JO, FR, GC, and NT performed staining and microscopy. MF, DM, SP-L, MD, EF, CR, FR, and NT performed the statistics, analyzed, and interpreted the data. MF, DM, SP-L, KS, FR, EF, and NT drafted the manuscript and designed the figures with contributions from the other authors. All authors read and approved the final manuscript.

### Conflict of Interest Statement

The authors declare that the research was conducted in the absence of any commercial or financial relationships that could be construed as a potential conflict of interest.

## Supplementary Material

Supplemental Figure 1 Cumulative incidence of local **(A)** and distant failure **(B)**, CSS **(C)** and OS **(D)** according to p16^INK4a^ expression (low: individual WS ≤ 6; high: individual WS > 6) in patients with CSCC treated with definitive CRT and BT.

Supplemental Table 1 Univariate and multivariate analyses of prognostic factors and combined PLK3/pT273 Caspase 8 variable in patients with CSCC.
