# Supplementary figures and images for: Association of Polo-Like Kinase 3 and PhosphoT273 Caspase 8 Levels With Disease-Related Outcomes Among Cervical Squamous Cell Carcinoma Patients Treated With Chemoradiation and Brachytherapy
Source: Front Oncol. 2019 Aug 14;9:742. doi: 10.3389/fonc.2019.00742 (PMC6702309; doi:10.3389/fonc.2019.00742)

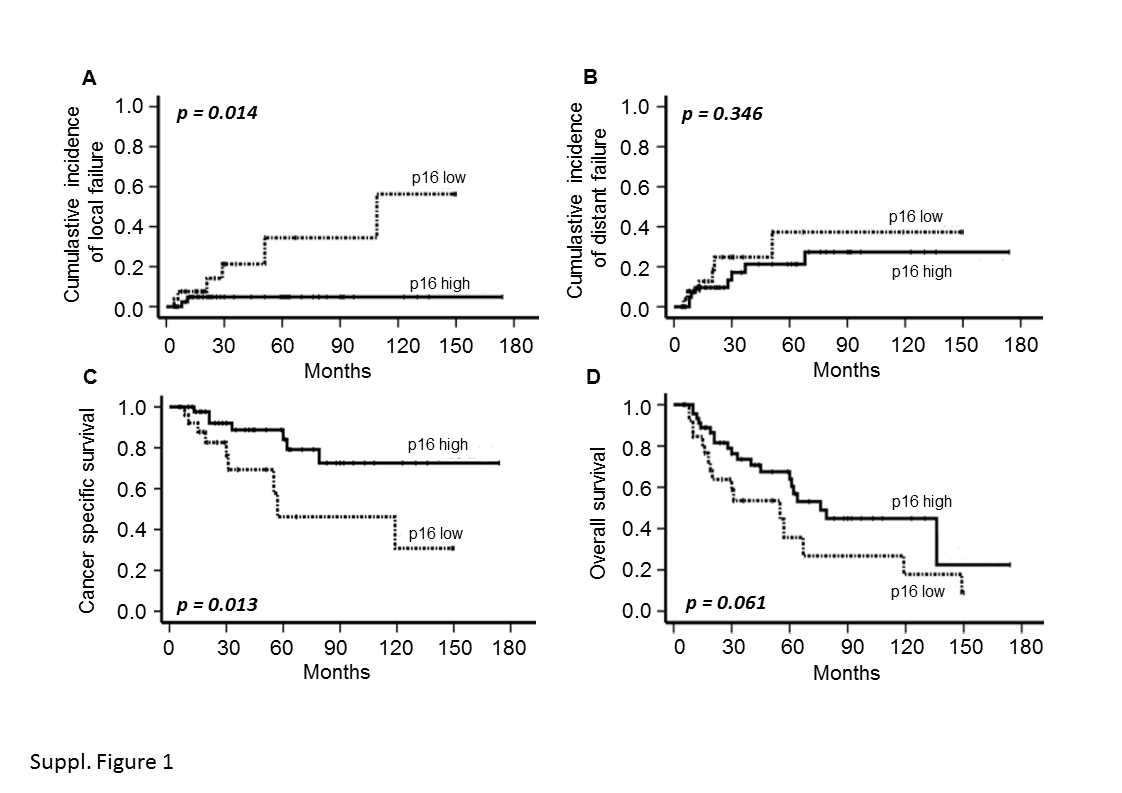

Supplement: Supplemental Figure 1 — Cumulative incidence of local (A) and distant failure (B), CSS (C) and OS (D) according to p16INK4a expression (low: individual WS ≤ 6; high: individual WS > 6) in patients with CSCC treated with definitive CRT and BT. [file Image_1.tif]
