# Supplementary material for: Association of Polo-Like Kinase 3 and PhosphoT273 Caspase 8 Levels With Disease-Related Outcomes Among Cervical Squamous Cell Carcinoma Patients Treated With Chemoradiation and Brachytherapy
Source: Front Oncol. 2019 Aug 14;9:742. doi: 10.3389/fonc.2019.00742 (PMC6702309; doi:10.3389/fonc.2019.00742)
Supplement: Supplemental Table 1 — Univariate and multivariate analyses of prognostic factors and combined PLK3/pT273 Caspase 8 variable in patients with CSCC. [file Table_1.pdf]

Supplemental Table 1. Univariate and multivariate analyses of prognostic factors and combined PLK3/pT273 Caspase 8 variable in patients with CSCC.

		Multivariate analyses			
			95 % Confidence interval (CI)		
	**Univariate p-value**	**Hazard ratio (HR)**	**lower**	**upper**	**p-value**
**Cumulative incidence of distant failure**					
T-stage (T1-2/T3-4)	**0.011**	1.28	0.26	6.36	0.756
FIGO (Ia-IIb/IIIa-IVb)	**0.008**	7.09	0.89	56.06	0.063
combined PLK3/pCasp 8	**0.005**	4.28	1.06	17.30	**0.041**
**Cancer-specific survival**					
T-stage (T1-2/T3-4)	**0.006**	1.36	0.15	11.87	0.777
FIGO (Ia-IIB/IIIa-IVb)	**0.017**	2.88	0.64	12.99	0.167
p16 (WS ≤ 6/>6)	**0.013**	1.75	0.60	5.03	0.299
combined PLK3/pCasp 8	**<0.001**	23.20	2.87	187.09	**0.003**
